# Tryptophan Metabolism Disorder-Triggered Diseases, Mechanisms, and Therapeutic Strategies: A Scientometric Review

**DOI:** 10.3390/nu16193380

**Published:** 2024-10-04

**Authors:** Xue Chen, Dong Xu, Jie Yu, Xu-Jiao Song, Xue Li, Yuan-Lu Cui

**Affiliations:** 1 Haihe Laboratory of Modern Chinese Medicine, Tianjin University of Traditional Chinese Medicine, Tianjin 301617, China; chenxue202210@163.com (X.C.); superdong1994@gmail.com (D.X.); yujie713812@163.com (J.Y.); songxujiao76@gmail.com (X.-J.S.); l111350538@163.com (X.L.); 2 State Key Laboratory of Component-Based Chinese Medicine, Research Center of Traditional Chinese Medicine, Tianjin University of Traditional Chinese Medicine, Tianjin 301617, China

**Keywords:** tryptophan metabolism, scientometric analysis, gut microbiota, COVID-19, depression

## Abstract

Background: Tryptophan is widely present in foods such as peanuts, milk, and bananas, playing a crucial role in maintaining metabolic homeostasis in health and disease. Tryptophan metabolism is involved in the development and progression of immune, nervous, and digestive system diseases. Although some excellent reviews on tryptophan metabolism exist, there has been no systematic scientometric study as of yet. Methods: This review provides and summarizes research hotspots and potential future directions by analyzing annual publications, topics, keywords, and highly cited papers sourced from Web of Science spanning 1964 to 2022. Results: This review provides a scientometric overview of tryptophan metabolism disorder-triggered diseases, mechanisms, and therapeutic strategies. Conclusions: The gut microbiota regulates gut permeability, inflammation, and host immunity by directly converting tryptophan to indole and its derivatives. Gut microbial metabolites regulate tryptophan metabolism by activating specific receptors or enzymes. Additionally, the kynurenine (KYN) pathway, activated by indoleamine-2, 3-dioxygenase (IDO) and tryptophan 2, 3-dioxygenase, affects the migration and invasion of glioma cells and the development of COVID-19 and depression. The research and development of IDO inhibitors help to improve the effectiveness of immunotherapy. Tryptophan metabolites as potential markers are used for disease therapy, guiding clinical decision-making. Tryptophan metabolites serve as targets to provide a new promising strategy for neuroprotective/neurotoxic imbalance affecting brain structure and function. In summary, this review provides valuable guidance for the basic research and clinical application of tryptophan metabolism.

## 1. Introduction

Tryptophan is an essential amino acid that can be obtained from dietary sources. Tryptophan metabolism is mainly concerned with three metabolic pathways: the kynurenine (KYN) pathway, the 5-hydroxytryptamine (serotonin, 5-HT) pathway, and the gut microbiota pathway. The KYN pathway predominantly occurs in immune and epithelial cells, accounting for about 95% of tryptophan metabolism. Tryptophan-2, 3-dioxygenase (TDO), indoleamine-2, 3-dioxygenase (IDO)1, and IDO2, the rate-limiting enzymes in the KYN pathway, fulfill crucial roles [1]. The 5-HT pathway, accounting for approximately 1–2% of tryptophan metabolism, occurs in central neurons and enterochromaffin cells (ECCs). Tryptophan can convert to 5-hydroxytryptophan (5-HTP) and further metabolize into 5-HT [2]. The gut microbiota (indole) pathway can directly convert tryptophan into indole and its derivatives, accounting for about 4–6% of tryptophan metabolism [3,4]. As a result, tryptophan metabolism plays a vital role in the development and progression of immune, nervous, and digestive system diseases [5]. An increasing number of studies focus on tryptophan metabolism, so there are some high-quality reviews [5,6,7]. However, by 31 December 2022, the Web of Science (WoS) database had witnessed a huge number of publications related to “tryptophan metabolism,” making it virtually impossible for scholars and researchers to be acquainted with all of them, let alone identify the major hotspots. To address this obstacle, this review employed scientometric methods to objectively and systematically summarize an overall outlook on the research trends in tryptophan metabolism, thereby enhancing researchers’ understanding of the tryptophan metabolism pathways and related diseases.

Scientometric analysis is a rigorous and popular approach for investigating and examining large amounts of scientific data [8]. It can be utilized to analyze advancements, identify strengths and weaknesses, uncover research gaps, and highlight research trends in various fields [9]. For example, Xu et al. used scientometrics to reveal the occurrence, development, and treatment of depressive disorder by analyzing induction factors, pathogenesis, comorbidity, animal models of depression, and therapy [10]. Xu et al. analyzed 100 of the most influential articles from the past 40 years to describe the evolution of hepatocellular carcinoma treatment using bibliometric citation analysis [11]. Based on scientometrics and other extensive studies, Ma et al. studied the regulatory importance of curcumin in various neural stem cell (NSC) activities, such as the differentiation, proliferation, and migration of NSCs, to treat mental and neurological diseases [12]. Therefore, we conclude that scientometric analysis is a hopeful strategy to explore and identify hotspots of tryptophan metabolism.

This article evaluates the current advancements in tryptophan metabolism research and employs scientometric analysis to discern emerging trends. We visually depict the annual publications, topics, keywords, and highly cited papers sourced from Web of Science spanning 1964 to 2022. Furthermore, we provide and summarize keywords that signify research hotspots and potential future directions, leveraging keyword co-occurrence analysis. Lastly, this review provides some solutions to address the primary challenges hindering the application of tryptophan metabolism in treating immune-mediated diseases. This review equips experts and newcomers alike with a panoramic view of their field, enabling them to locate novel research avenues and devise future research plans in an intuitive, visually engaging manner, significantly enhancing researchers.

## 2. Data and Methods

### 2.1. Data Retrieval and Download

**Figure 1:**
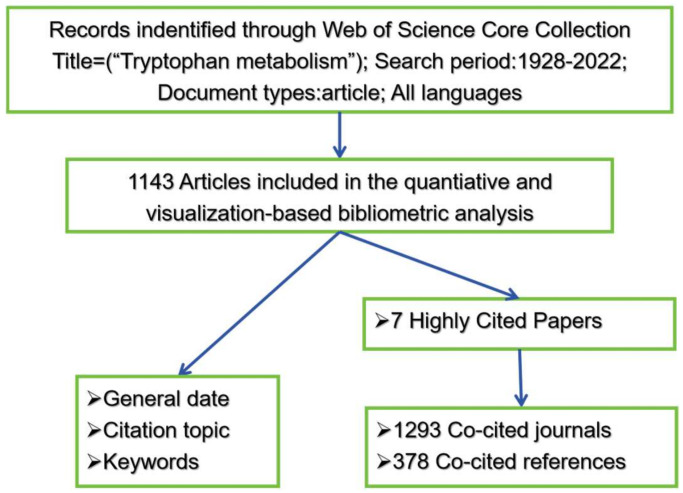
Flowchart for the data retrieval and process.

The term “tryptophan metabolism” as a title was searched in the Web of Science Core Collection database (WOS) until 7 July 2023. Articles up to 31 December 2022 were refined and used for scientometric analysis. The downloaded contents include 1143 articles, 7 highly cited articles, and their 378 references and 1293 citations. The methodology for retrieving and gathering data is illustrated in Figure 1. The record contents contain full records and cited references in plain text file format.

### 2.2. Data Analysis and Visualization

Data analysis and visualization were used to present the current and future study of tryptophan metabolism. In brief, keyword analysis was performed using bibliometrix (version: 3.1.4) and ggplot2 (version: 3.4.1), focusing mainly on hotspot keyword examination, keyword clustering evaluation, and keyword evolution exploration [13]. Meanwhile, the evolution of the highly cited papers is explored by visualizing their references and citations. Topic analysis is shown by summarizing citation topics.

## 3. Scientometric Studies

**Figure 2:**
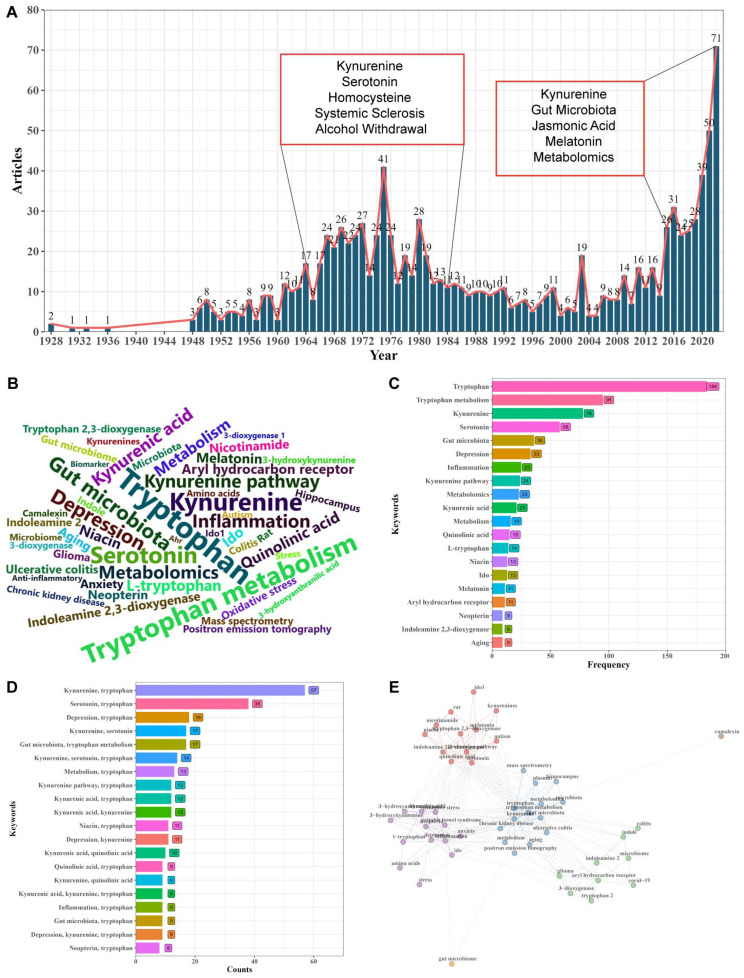
Keyword analysis. (**A**) Number of publications by year. (**B**) Word cloud. (**C**) Top 20 keywords. (**D**) Top 15 co-occurrence keywords. (**E**) Keyword co-occurrence network.

A total of 1143 papers about tryptophan metabolism were obtained from WOS, covering 1928 to 2022. Figure 2A depicts an increasing publication number of tryptophan metabolism. In 1928, Robson published two articles on the formation of kynurenic acid (KYNA) from tryptophan, shedding light on tryptophan metabolism [14,15]. From 1964 to 1984, there is a small peak in publication number, with a focus on “Kynurenine”, “Serotonin”, “Homocysteine”, “Systemic sclerosis”, and “Alcohol withdrawal”. Furthermore, from 2015 to 2022, the analysis reveals a significant growth trend, with a focus on “Kynurenine” and “Gut microbiota”, “Jasmonic acid”, “Melatonin”, and “Metabolomics”. Publications increased from eight in 2008 to seventy-one in 2022, with nearly 56.80% published in the last five years. These studies provide a crucial foundation for understanding the physiological function of tryptophan metabolism and its relationship with diseases.

### 3.1. Keyword Analysis

According to the scientometric theory, keywords can provide insights into the core area of the research domain [16]. In this review, keyword visualization is used to present high-frequency keywords (Figure 2B,C), the top 15 co-occurrence keywords (Figure 2D), and the keyword co-occurrence network (Figure 2E). Based on the results of the word cloud and the top 20 keywords, the most relevant keywords are “Tryptophan”, followed by “Tryptophan metabolism”, “Kynurenine”, “Serotonin”, and “Gut microbiota”, indicating the significance of tryptophan metabolism and its metabolic pathway. “Depression” and “Inflammation” closely follow, suggesting that tryptophan metabolism may play a role in treating inflammation-induced depression. The presence of “Kynurenine”, “Kynurenine pathway”, and “Kynurenine acid” highlights the importance of KYN metabolism, which is consistent with research findings of tryptophan metabolism. Over 95% of tryptophan is metabolized via the KYN pathway to synthesize various bioactive compounds.

The top 15 co-occurrence keywords of tryptophan metabolism reveal that the combinations “kynurenine, tryptophan” (18.75%) and “serotonin, tryptophan” (12.50%) appear most frequently, followed by combinations such as “depression, tryptophan”, “kynurenine, serotonin”, and “gut microbiota, tryptophan metabolism”, which have appeared more than 15 times. These associations demonstrate the close relationship between tryptophan metabolism and its three metabolic pathways.

The keyword co-occurrence network divides keywords into four clusters based on a clustering algorithm, with different colors representing different clusters. Betweenness centrality is often used to quantify the importance of a node in a network. Based on the betweenness centrality of keywords, this review summarized the main topic of four clusters: cluster 1 (tryptophan metabolism pathway), cluster 2 (gut microbiota), cluster 3 (COVID-19 and glioma), and cluster 4 (inflammation-induced depression) (Table 1). Cluster 1 (red) includes keywords such as “tryptophans”, “serotonin”, “quinolinic acid”, “kynurenine pathway”, “indoleamine-2, 3-dioxygenase”, etc. From this cluster, it is concluded that there are primarily publications focusing on the KYN and serotonin pathways. Cluster 2 (green) includes “tryptophan metabolism”, “kynurenine”, “metabolism”, “gut microbiota”, “metabolomics”, etc. This cluster indicates that the gut microbiota has an essential influence on tryptophan metabolism. Specifically, the gut microbiota can affect the KYN metabolism to improve diseases. Cluster 3 (purple) includes “COVID-19”, “glioma”, “aryl hydrocarbon receptor”, “microbiome”, “colitis”, etc. Regarding keyword centrality, the current research hotspots in this cluster revolve around COVID-19 and glioma. Cluster 4 (blue) includes “inflammation”, “depression”, “kynurenic acid”, “irritable bowel syndrome”, “neopterin”, etc. It can be seen that tryptophan metabolism regulates inflammation-induced depression, which may become a research hotspot. This information provides further insights into the research trends and relationships within the field of tryptophan metabolism.

### 3.2. The Highly Cited Papers

Citation is a widely used metric in scientific research analysis, as it quantifies the relative influence of scientific papers within a specific field [17,18]. Highly cited papers are often considered high in quality, influence, and innovation.

Table 2 provides details of the first seven highly cited original articles about tryptophan metabolism, published between 2016 and 2022. Notably, the study by Bruno Lamas et al., which investigates the complex interactions between the host and intestinal microbiota and the impact of CARD9 on colitis, received the highest number of citations (760). It is revealed that colitis occurrence is influenced by CARD9, which alters the tryptophan in the gut microbiota, making it an aryl hydrocarbon receptor (AhR) ligand. CARD9 promotes colitis recovery by increasing the production of IL-22. Tryptophan cannot be metabolized into metabolites that act as ligands for the AhR in mice lacking CARD9. However, when mice are treated with AhR agonists or administered tryptophan-metabolizing lactic acid bacteria, intestinal inflammation is alleviated [19].

Furthermore, the second and sixth articles explore the relationship between tryptophan metabolism and inflammatory bowel disease (IBD) [20,24]. In patients with active IBD, increased tryptophan metabolites, specifically higher levels of quinolinic acid (QA), indicate a high level of tryptophan catabolism. The deficiency of tryptophan might be a factor in the development or exacerbation of IBD. Another study focuses on Fuzhuan Brick Tea Polysaccharide (FBTP) and its effect on ulcerative colitis (UC) about gut microbiota-derived tryptophan metabolism. FBTP may alleviate UC by regulating the gut microbiota, repairing the intestinal barrier, and promoting microbial metabolism. These studies shed light on the impact of tryptophan metabolism on IBD and its therapeutic mechanisms, leading to subsequent follow-up studies in this area. Overall, these highly cited articles have significantly contributed to our understanding of tryptophan metabolism and its implications in colitis and IBD, providing valuable insights for further research. Indeed, microbial changes in tryptophan metabolism have been found to be associated with gastrointestinal dysfunction [21]. Reduced abundance of the bile metabolizing species of Bifidobacterium and Blautia bacteria is observed in a mouse model of autism. Additionally, there is a deficiency in the bacterial metabolism of bile moieties in the intestine of these mice. Gut microbiota changes are associated with significant gastrointestinal distress and reduced sociability in BTBR mice. This research highlights the critical role of the gut microbiota and its importance in tryptophan metabolism and gastrointestinal function. The findings suggest that microbial composition and function disruptions will contribute to gastrointestinal dysfunction and associated symptoms. Understanding these relationships may help develop novel therapeutic strategies for treating gastrointestinal disorders and related conditions.

The analysis of fourth-, fifth-, and seventh-ranked highly cited articles confirms that tryptophan metabolites can function as ligands to treat various diseases by regulating AhRs. These studies demonstrate the potential of using tryptophan metabolites to improve conditions such as alcohol-induced liver injury, enhance learning and memory behavior, and inhibit tumor growth in the bone marrow [22,23,25]. AhRs are cytoplasmic transcription factors initially known for their role in xenobiotic detoxification and have a crucial role in tryptophan metabolism by interacting with tryptophan metabolites. As a result, these receptors have become attractive therapeutic targets in autoimmune diseases, cancer, neurodegenerative diseases, and intestinal disorders [4].

**Figure 3:**
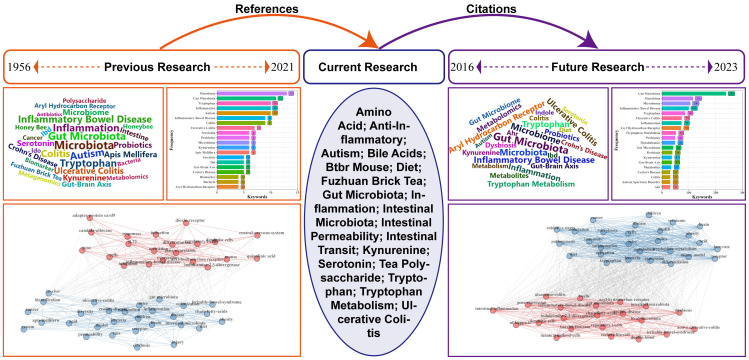
The previous, current, and future keyword analyses of highly cited papers.

Figure 3 provides a keyword analysis of highly cited articles and their references and citations. The left figure (orange) analyzes references of highly cited papers from 1956 to 2021, reflecting the historical research focus on tryptophan metabolism. The right (purple) analyzes citations of highly cited papers from 2016 to 2023, providing insights into future research trends. The middle (blue) summarizes the critical keywords extracted from the highly cited papers, bridging previous and future research. Exploring and summarizing previous theories provides an essential guide for future studies of tryptophan metabolism.

The comparison of high-frequency keywords of references and citations reveals that “intestinal microbes” and “inflammatory bowel disease” have consistently been the focal points of research on tryptophan metabolism. Tryptophan metabolites are crucial as mediators of communication between the host and intestinal microbiota. Studies have demonstrated that the microbiota directly or indirectly controls the tryptophan metabolic pathways, and numerous tryptophan metabolites drive the crosstalk between the host and its microbiota [26,27]. Disorders of tryptophan metabolism are significantly associated with various clinical features of IBD, with tryptophan and its metabolites affecting perianal involvement, disease activity, severity of inflammation, and extraintestinal manifestations of IBD [20,28,29,30]. AhR is a ligand-activated transcription factor involved in various cellular processes and immune regulation. It regulates intestinal homeostasis by promoting the release of cytokines such as IL-22, IL-17, and IL-6 and also participates in adaptive immunity via modulation of immune cell responses. Cyp1A1, a protein in the cytochrome P450 family, forms a feedback loop in AhR signaling by inactivating various AhR ligands [31]. Moreover, “probiotics” are substrates that have a beneficial effect on health and are selectively utilized by the microorganisms of the host. Probiotics are mainly sourced from indigestible dietary fibers such as oligosaccharides, fructans, and galactose [32]. Supplementation of probiotics and prebiotics may hold therapeutic potential in improving abnormal KYN pathway metabolism, further emphasizing their importance in the context of tryptophan metabolism research [33].

The comparison of the keyword co-occurrence network of references and citations reveals a shift of research focus over time. In the previous research, two clusters emerged, highlighting the relationship between tryptophan metabolism and immunity and tryptophan metabolism and disease. These clusters represent broader research themes in the field. However, two clusters have also emerged, indicating toward future research. The first cluster focuses on linking tryptophan metabolism to IBD, suggesting a growing interest in understanding how tryptophan metabolism is involved in initiating and progressing IBD. This cluster likely represents a more targeted investigation into the function of tryptophan metabolism in IBD. The second cluster centers around tryptophan metabolism and tumors. Researchers are delving into the mechanisms by which tryptophan and its metabolites influence tumor biology, potentially paving the way for developing novel therapeutic strategies. The shift in the keyword co-occurrence network reflects a transition toward more specific and detailed research investigations in tryptophan metabolism, particularly on IBD and tumors.

### 3.3. Topic Analysis

**Figure 4:**
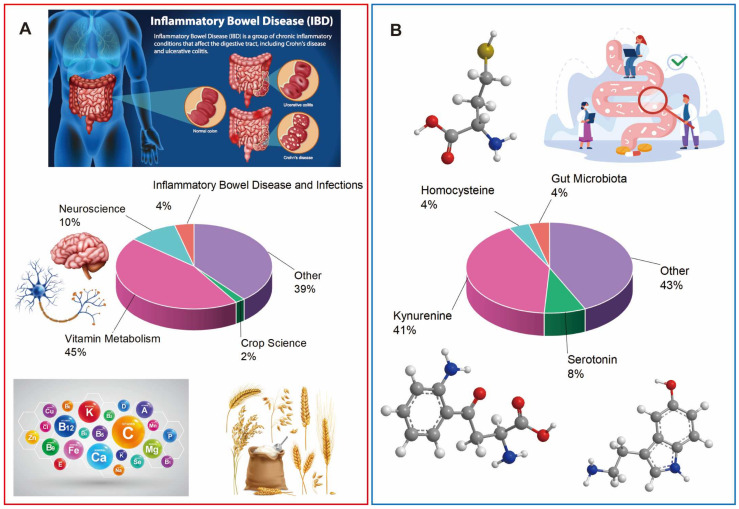
Topic analysis of tryptophan metabolism. (**A**) Citation topics meso (among the other 39%, 94 topics are included in the “citation topics meso”, such as Dairy and Animal Sciences 2%, Sleep Science and Circadian Systems 1%, Rheumatology 1%, etc.). (**B**) Citation topics micro (among the other 43%, 187 topics are included in the “citation topics micro”, such as Melatonin 1%, Broiler 1%, Systemic Sclerosis 1%, etc.).

The 1143 papers retrieved from WOS were identified and downloaded with their “Citation Topic Meso” and “Citation Topic Micro” data, and the macro and micro citation topics were detailed separately for the first five topics. Figure 4A is the meso view of the citation topic. “Vitamin Metabolism”, “Neuroscience”, and “Inflammatory Bowel Diseases & Infections” belong to the macro topic “Clinical & Life Sciences”, in which vitamin metabolism accounts for up to 45%. The second category, namely “Crop Science” and “Dairy & Animal Sciences”, falls under the umbrella of “Agriculture, Environment & Ecology”. It is indicated that tryptophan metabolism plays a crucial role in the human body and holds significant implications in agriculture and ecology. In Figure 4B, the top five micro topics are depicted, namely “Kynurenine”, “Serotonin”, “Homocysteine”, “Gut Microbiota”, and “Melatonin”, which belong to the meso topics “Vitamin Metabolism”, “Neuroscience”, “Vitamin Metabolism”, “Inflammatory Bowel Diseases & Infections”, and “Sleep Science & Circadian Systems”, respectively. These meso topics fall under the broader “Clinical & Life Sciences” category. In the micro topic analysis, “Kynurenine” had the highest percentage at 41%, followed closely by “Gut Microbiota” and “Melatonin”, suggesting the three metabolic pathways. The KYN pathway is crucial in immune responses, regulating inflammation and excitatory neurotransmission in vivo. The serotonin pathway may regulate adaptive responses to environmental changes, affecting cognition, eating behavior, and sleep. The gut microbiota can convert tryptophan directly into different indole molecules. This conversion helps maintain intestinal homeostasis by regulating the expression of pro-inflammatory and anti-inflammatory cytokines [5]. Overall, tryptophan metabolism has broad implications in various pathophysiological processes, and thus, understanding the mechanisms and targets of tryptophan metabolism pathways is necessary to provide valuable insights for developing targeted therapeutics for related diseases.

## 4. Major Finding of Scientometric Results

Based on the results of scientometric analysis, research hotspots are obtained from tryptophan metabolism. The hotspot content mainly includes tryptophan metabolism pathways, gut microbiota, COVID-19 and glioma, and inflammation-induced depression. Tryptophan metabolism disorder-triggered diseases, their mechanisms, and their treatment will be reviewed in conjunction with research hotspots.

### 4.1. Mechanism of Tryptophan Metabolism

#### 4.1.1. Tryptophan Metabolism Pathway

**Figure 5:**
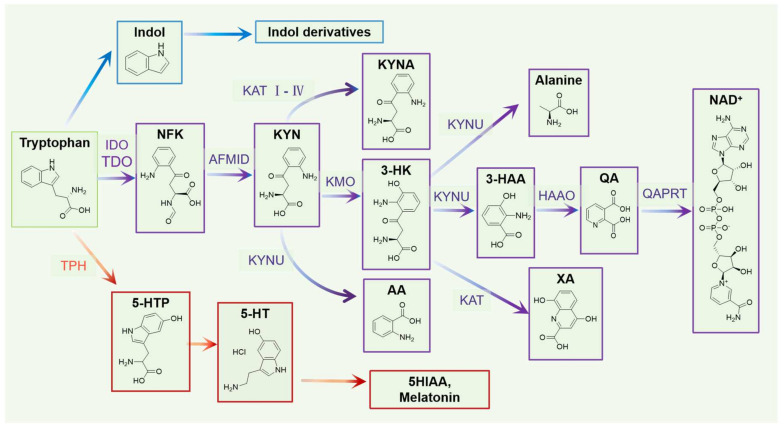
Flowchart of the three tryptophan metabolic pathways (NFK, N-formyl kynurenine; KYN, kynurenine; KYNA, kynurenic acid; 3-HK, 3-hydroxy-kynurenine; XA, xanthurenic acid; AA, anthranilic acid; 3-HAA, 3-hydroxy anthranilic acid; QA, quinolinic acid; NAD+, N-methyl-D-aspartic acid; 5-HT, 5-hydroxytryptamine; 5-HTP, 5-hydroxytryptophan; 5HIAA, 5-hydroxyindole-3-acetic acid).

Tryptophan is one of the human essential amino acids. Through intestinal epithelial cells, tryptophan can be absorbed from food and enter the blood. Tryptophan metabolism helps regulate physiological functions and participates in plasma protein renewal and synthesis of nicotinic acid and melatonin. Tryptophan metabolism mainly includes the KYN, 5-HT, and indole pathways (Figure 5).

KYN metabolism is the main pathway in which >95% of tryptophan is converted into multiple active biological compounds. KYN metabolism mainly occurs in the liver. The three rate-limiting enzymes in the KYN pathway, namely tryptophan-2, 3-dioxygenase (TDO), indoleamine-2, 3-dioxygenase (IDO) 1, and IDO2, play a key role. TDO is present in the liver and brain, whereas IDO is not expressed in most healthy human tissues. TDO is mainly regulated by glucocorticoids and tryptophan levels, whereas IDO is induced by a variety of pro-inflammatory mediators, including interleukin-2 (IL-2), interferon-gamma (IFN-γ), and tumor necrosis factor-alpha (TNF-α) [34]. KYN can positively affect the expression and activity of IDO [35]. The metabolism of KYN occurs in four steps. First, tryptophan is degraded to N-formyl kynurenine (NFK) by TDO and IDO. NFK is metabolized to KYN by arylformamidase (AFMID). Second, KYN is catalyzed by KYN aminotransferases (KAT I-IV), kynurenine-3-monooxygenase (KMO), and kynureninase (KYNU) to three parts—kynurenic acid (KYNA), 3-hydroxy-kynurenine (3-HK), and anthranilic acid (AA), respectively. KYNA is one of the ligands of AhR that noncompetitively binds to the N-methyl-D-aspartate (NMDA) glutamate receptor to inhibit excitotoxicity and neuroinflammation [36]. Third, 3-HK is converted to 3-hydroxy anthranilic acid (3-HAA), alanine, and xanthuric acid (XA); 3-HK is a neurotoxic compound that can produce nanomolar concentrations of free radicals that affect neuronal degeneration and apoptosis. Fourth, 3-HAA can be converted into neurotoxic QA by 3-hydroxyanthranilate-3, 4-dioxygenase (HAAO). QA has been shown to exert neurotoxic effects, particularly on astrocytes, through the selective activation of NMDA receptors. NMDA is a glutamate receptor involved in synaptic transmission and has been implicated in various neurological disorders. Furthermore, QA is further transformed to NAD+ through the function of QA phosphoribosyltransferase and NAD synthase. NAD+ is crucial for cellular energy metabolism, as it is an essential coenzyme in various metabolic pathways, including oxidative phosphorylation and glycolysis. NAD+ plays a vital role in transferring electrons during cellular respiration, which is necessary for generating ATP. Besides its role in energy metabolism, NAD+ is also involved in other cellular processes. It is a co-factor for enzymes involved in DNA repair, cell division, and mitochondrial functions. NAD+ is an essential regulator of cellular processes, and its levels can impact various physiological and pathological conditions [37]. 

About 1–2% tryptophan is catalyzed by TPH to produce 5-HTP and then 5-HT. The latter is a phylogenetically conserved monoamine neurotransmitter in the CNS, which regulates some brain functions, such as appetite, sleep, and emotion [38]. The latter is also a multifunctional signaling molecule of the gastrointestinal tract, which affects intestinal motility, secretion, movement, vasodilation, and nutrient absorption [39]. In addition, as a peripheral hormone, 5-HT can play an immune role by regulating the secretion of cytokines, activating the 5-HT receptor, starting T cells, and recruiting neutrophils [40]. Over 95% of 5-HT is synthesized in TPH1 of enterochromaffin cells (ECCs), and a small part can be synthesized in the brain periphery. However, 5-HT synthesized in the intestine cannot modulate the function of the CNS or cross the blood–brain barrier. The signal molecule 5-HT is transformed to 5-HIAA (5-hydroxyindole-3-acetic acid), which regulates emotions, anxiety, and behavior and is a potential biomarker for monitoring the development of many neurological and mental disorders [41]. Additionally, 5-HT can be metabolized into melatonin, further regulating the sleep awakening cycle.

Additionally, the gut microbiota converts a tiny amount of tryptophan into indole-3-acetic acid (IAA), indole acrylic acid, indole-3-acetaldehyde (3-IAld), indole-3-propionic acid (IPA), tryptamine, etc. These metabolites help regulate gastrointestinal function, inflammation, the immune system, and anti-oxidation. Indole can reduce intestinal inflammation and positively impact the homeostasis of the gastrointestinal tract and liver [42]. Through immune-mediated mechanisms, IPA may help regenerate and restore sensory axons [43]. These metabolites act as ligands that bind to AhR, thus participating in immune regulation and various cellular processes. However, over-activation of the AhR signaling pathway may negatively affect cells. CYP1A1 is a cytochrome P450 enzyme that can regulate the activity of the AhR signaling pathway by metabolizing and inactivating the AhR ligand. This regulation forms a feedback loop that can limit the cellular response caused by excessive activation of AhR signal transduction [31]. In addition, IL-4I1 can metabolize L-tryptophan into indole-3-neneneba pyruvate in some cells and produce IAA, indole-aldehyde (IAld), and ILA. By activating AhR, these metabolites can regulate immune cell function and inflammatory response and affect tumors’ growth, survival, and migration ability [44].

#### 4.1.2. Gut Microbiota

The gut microbiota can produce molecules capable of interacting with host physiology and triggering responses at the local and distal levels, acting as a virtual endocrine organ [45]. The communication between intestinal microbes and the host is facilitated by three distinct categories of metabolites: short-chain fatty acids, bile acids, and tryptophan metabolites. In this section, the impact and mechanism of intestinal microbiota on tryptophan metabolism will be highlighted.

**Figure 6:**
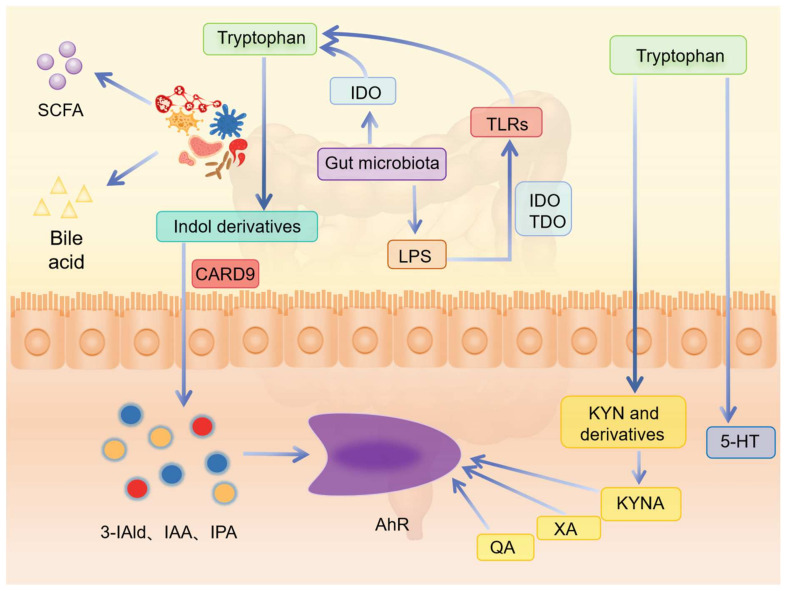
Influence of host intestinal flora on tryptophan metabolism (SCFA, short chain fatty acid; IDO, indoleamine-2, 3-dioxygenase; TDO, tryptophan-2, 3-dioxygenase; TLRs, toll-like receptors; CARD9, caspase recruitment domain-containing protein 9; LPS, lipopolysaccharide; 3-IAld, indole-3-acetaldehyde; IPA, indole-3-propionic acid; IAA, indole-3-acetic acid; AhR, aryl hydrocarbon receptor; KYN, kynurenine; KYNA, kynurenic acid; QA, quinolinic acid; XA, xanthurenic acid; 5-HT, 5-hydroxytryptamine).

In the gut, the microbiota directly or indirectly controls the metabolic pathway of tryptophan (Figure 6). On the one hand, intestinal microbes can instantly transform tryptophan into indole and its derivatives and regulate intestinal permeability, inflammation, and host immunity. At the same time, it affects the balance between 5-HT synthesis and the tryptophan degradation pathway [26]. In normal control mice, a significant increase in serum levels of 5-TH were observed in comparison to sterile feeding mice [46,47]. Notably, intestinal microorganisms have been found to elevate TPH1 expression in the colon, which may represent a pivotal mechanism underlying the regulation of intestinal 5-HT homeostasis [48]. Additionally, in germ-free mice, the KYN pathway is inhibited, resulting in decreased tryptophan levels. However, upon supplementation with intestinal flora, tryptophan metabolism via the KYN pathway is significantly augmented [49]. On the other hand, some metabolites of intestinal microorganisms regulate tryptophan metabolism by activating specific receptors or enzymes. LPS produced by intestinal microbes can start tryptophan metabolism by activating toll-like receptors (TLRs), while butyrate can regulate tryptophan metabolism by inhibiting IDO activity [26,33]. Several studies have established a connection between TLRs and metabolic changes within the KYN pathway [50]. Variations in the intestinal microbiota can lead to abnormal activation of TLRs, thereby potentiating the activity of the KYN pathway. Furthermore, alterations in IDO1 activity have been suggested to mediate the regulatory role of TLRs on the KYN pathway [51]. Intriguingly, certain gut bacteria possess enzymes that exhibit homology to key enzymes involved in the KYN pathway, enabling them to produce a diverse array of KYN pathway metabolites [52].

The KYN pathway is the primary metabolic mode of tryptophan, and the critical rate-limiting enzymes IDO and TDO affect it [53]. IDO has immune responsiveness and is regulated by inflammatory mediators, significantly induced by INF-γ. The gut microbiota controls host intestinal tryptophan metabolism by regulating IDO1 activity, thus affecting the produced metabolites, such as KYN and its downstream products (QA, KYNA) [33,54]. KYN, QA, and KYNA metabolites regulate neurotransmission, inflammation, and immune responses. QA is produced by microglia and can selectively activate NMDA receptors to exert neurotoxicity on astrocytes. However, KYNA is produced by astrocytes and selectively antagonizes NMDA receptor as a neuroprotection. The ratio of KYNA to QA suggests a balance between neurotoxicity and neuroprotection and is widely concerned about diseases of neurological impairment such as depression and epilepsy. In conclusion, the intestinal microbiota exerts an essential influence on intestinal tryptophan metabolism and interacts with the host through the produced metabolites to regulate the physiological functions of the host.

In recent years, it has been found that tryptophan metabolism is one of the potential avenues for bidirectional gut–brain communication [55]. Research has uncovered molecular pathways in which enteroendocrine cells (EECs) modulate intestinal and vagal neuronal pathways in response to microbial signals [56]. Notably, germ-free rats colonized with indole-producing bacterial species Escherichia coli exhibited vagus nerve activation and heightened anxiety-like behavior, in contrast to germ-free rats incapable of producing indole. People with gut microbiota that readily produce indole are more prone to develop anxiety and mood disorders [57]. Furthermore, Xie et al. have explored the role of tryptophan in Alzheimer’s disease from the perspective of gut–brain axis crosstalk with microglia and astrocytes. Microbiota-derived tryptophan and its metabolites enter the central nervous system to control microglial activation, which subsequently influences astrocyte function, modulating the progression of Alzheimer’s disease [55]. By understanding and modulating these microbial–neuronal interactions, we may uncover novel therapeutic strategies for managing anxiety, mood disorders, and neurodegenerative diseases like Alzheimer’s. Further, it has been found that bacterial tryptophan metabolites can help develop new drugs for treating gut–brain axis disorders [57,58]. 

### 4.2. Diseases Related to Tryptophan Metabolism

#### 4.2.1. COVID-19 and Glioma

Since the discovery of the immunomodulatory effect of tryptophan metabolism, more and more evidence supports that it can treat inflammation, tumor, and other diseases [59,60,61,62]. In recent years, the role of tryptophan metabolism in the immunomodulation of COVID-19 and glioma has become a research hotspot.

**Figure 7:**
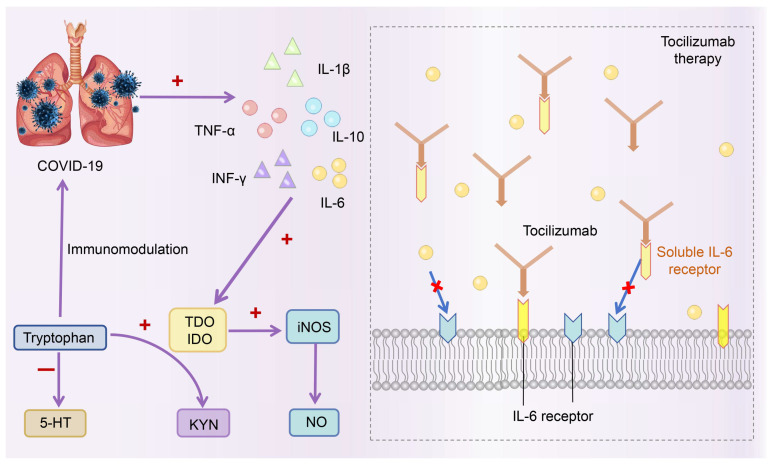
Tocilizumab regulates tryptophan metabolism in treating COVID-19. The “+” in the figure represents promotion, and the “-” represents inhibition. (TDO, tryptophan-2, 3-dioxygenase; IDO, indoleamine-2,3-dioxygenase; KYN, kynurenine; 5-HT, 5-hydroxytryptamine; IL-6, interleukin-6; IL-1β, interleukin-1β; IL-10, interleukin-10; TNF-α, tumor necrosis factor; INF-γ, interferon-γ; iNOS, inducible nitric oxide synthase; NO, nitric oxide).

COVID-19 is a viral disease caused by Severe Acute Respiratory Syndrome Coronavirus 2 (SARS CoV-2), which is closely contacted by droplet spray and respiratory transmission. The severity of COVID-19 mainly depends on activating the immune system and secreting inflammatory factors, causing systemic damage to various organs [63,64,65]. In 2019, COVID-19 broke out, and the medical community was widely concerned about the effects of tryptophan metabolism on the human body [66,67,68]. Tryptophan catabolism is a powerful immune regulatory pathway to fight against excessive inflammatory reactions, which will produce a wide range of immunoreactive metabolites. Clinical studies have shown that patients with severe COVID-19 have a selective increase in the KYN pathway of tryptophan metabolism [69]. Metabonomic analysis of serum samples from patients with COVID-19 shows that the serum tryptophan level decreased, and intermediate metabolite (KYN: KYNA) increased compared with the healthy control group [70,71]. Compared with the healthy control group, the level of 5-HT in the blood of patients with severe COVID-19 is lower, which indicates that during SARS-CoV2 infection, the KYN pathway explicitly promotes the utilization of tryptophan [70]. In addition, IDO1 and its metabolic products of the KYN pathway are being actively investigated as immunomodulatory agents [72,73,74]. Under physiological conditions, IDO1 is expressed in monocytes and dendritic cells in organs outside the liver and is activated by inflammatory cytokines. It has been proven that there is a close correlation between inflammation driven by IL-6 and lack of IDO1 activity, thereby impairing the role of its immune homeostasis regulator (Figure 7) [75]. Tocilizumab enhanced the IDO1-mediated conversion of tryptophan to its metabolite KYN by neutralizing IL-6 bioactivity. Increased circulating KYN levels help control pulmonary hypertension and inflammation and promote immunomodulation in patients with COVID-19 [69]. Under highly inflammatory conditions, aberrant secretion of IL-6 triggers enzymatic IDO1 hydrolysis, leading to accelerated hydrolysis and disruption of effective immunomodulatory mechanisms. Excessive degradation of IDO1 protease induces autoimmune diabetes and contributes to autoimmune responses against pancreatic autoantigens [76]. Regarding innate immunity, metabolites produced by IDO1 inhibit the secretion of type I IFN through the activation of AhR [77,78]. In severe COVID-19 patients, the activation of AhR by IDO1-derived tryptophan metabolites acts as a biological defense mechanism [70].

Glioma is the most common primary malignant tumor of the CNS (80%), and there is currently no breakthrough treatment method [79]. Exploring new therapies for glioma, especially immunotherapy, has gradually become a research hotspot. The changes in tryptophan metabolism can regulate the immune function of glioma by affecting the tryptophan immune microenvironment and tumor cells [80]. The catabolic product of tryptophan, KYN, can induce cancer cell invasion and immune suppression of the tumor microenvironment by binding to AhR [81,82,83,84]. IDO and TDO levels are crucial to tryptophan metabolite changes in glioma. The effectiveness of immune checkpoint inhibitors is inversely correlated to the elevated levels of IDO in glioma, suggesting that higher IDO levels are related to poorer treatment outcomes. The poor prognosis of glioma indicates an up-regulation of TDO2 expression [85,86]. DO1 is widely expressed in glioma, which can predict the poor prognosis of glioma patients, and TDO can promote tumor progression [84,87,88]. IDO and TDO positively correlate with glioma grading and may promote the migration and invasion of glioma cells through the KYN/AhR/APQ4 signaling pathway [89]. KYNA, tryptophan catabolite, can induce cancer cell invasion and inhibit tumor microenvironment by binding with AhR [81,90]. Activating the AhR can hinder the ability of macrophages and T cells to exert anti-tumor effects [90]. The dynamics of the KYN enzyme can cause or promote CNS disease. For example, tryptophan has been found to accumulate significantly in the cerebrospinal fluid of patients with glioma [91]. In contrast, the high expression of IDO and other enzymes in glioma cells increases, leading to tryptophan depletion and metabolite accumulation in cells and the microenvironment. Lastly, IDO and TDO promote the development of glioma while suppressing anti-tumor immune responses in the microenvironment. Additionally, Zhang et al. found that tryptophan metabolism-related genes (TrMGs) are closely related to the clinicopathological and immunological characteristics of glioma. With its strong prognostic predictive ability for glioma patients, TrMRS is widely used. Higher levels of TrMRS represent more active tryptophan metabolism, and conversely, lower levels predict more immune infiltration and immunosuppression [92]. These indicate a better comprehension of tryptophan-related metabolism [93,94].

#### 4.2.2. Inflammation-Induced Depression

**Figure 8:**
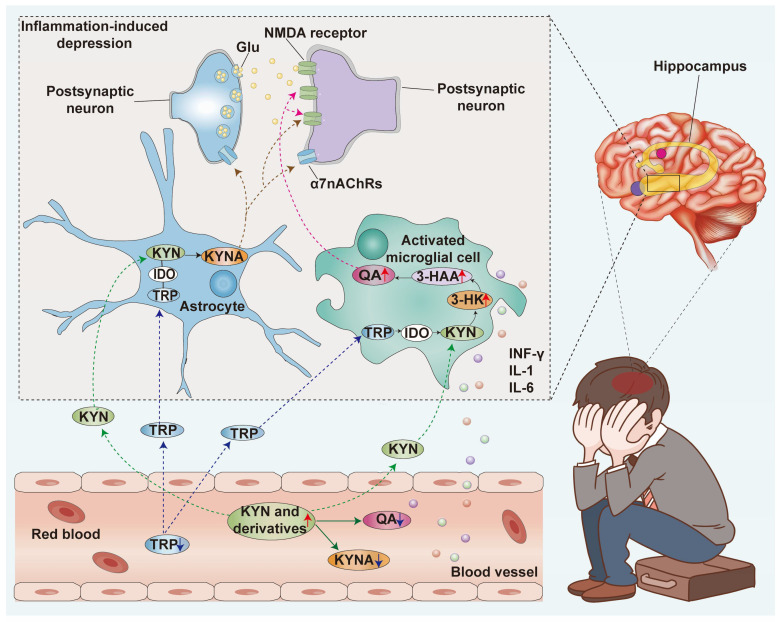
The link between the KYN pathway, intestinal inflammation, and inflammation-induced depression. The symbol “↑” in the figure represents an increase, while “↓” represents a decrease. (TRP, tryptophan; Glu, glutamic acid; IDO, indoleamine-2, 3-dioxygenase; KYN, kynurenine; INF-γ, interferon-γ; TNF-α, tumor necrosis factor; KYN, kynurenine; KYNA, kynurenic acid; 3-HK, 3-hydroxy-kynurenine; 3-HAA, 3-hydroxy anthranilic acid; QA, quinolinic acid; NMDA receptor, N-methyl-D-aspartate receptor).

As is well known, immune-mediated inflammatory diseases coexist with depression [95,96]. Research has shown that the KYN metabolic pathway has been implicated in the development of depression caused by inflammation by affecting the brain’s glutamate receptor [97,98]. Recently, researchers have begun to focus on the metabolic enzymes and metabolites of KYN in the study of inflammation-induced depression. Inflammatory cytokines and peripheral inflammatory stimuli such as TNF, IFN-γ, and LPS convert tryptophan into KYN by activating IDO. During the depression, enzymes IDO and TDO are over-activated and can be used to treat depression [99]. Inhibiting the activation of the KYN metabolic pathway in mice by inhibiting IDO can reverse the depression-like behavior induced by LPS [100]. Excessive production of pro-inflammatory cytokines in depression induces IDO enzymes and promotes the KYN pathway, thereby reducing the activation of the 5-HT pathway [101]. In addition to IDO and TDO, other KYN metabolic enzymes are associated with depression, including KAT activity, KMO activation, KMO, and KAT III single-nucleotide polymorphism [102,103]. In addition, a neuroprotective compound is metabolite KYNA, while its downstream product QA is a neurotoxic compound. KYNA is an agonist of the NMDA receptor, while QA is an NMDA receptor antagonist. Blocking the NMDA receptor can reduce depression-like behavior induced by lipopolysaccharide [104]. In humans, plasma–KYN metabolites may mediate inflammation-related depression symptoms through CNS-KYN metabolites, and CNS-KYN metabolites can serve as intervention targets for peripheral KYN metabolism, inflammation, and KYN transport to the brain [105]. Studies have found that QA and KYNA, metabolites of the KYN pathway, have neural activity, and they are hot molecules in the mechanism of inflammation-induced depression in recent years (Figure 8) [106]. Inflammation-induced depression is hypothesized to be related to a reduction in the KYNA/QA ratio, leading to an imbalance in neuroprotection and neurotoxicity that potentially affects brain structure and function [107]. QA mainly exists in the forebrain. In the CNS-KYNA metabolism process, the rate of 3-HAA converting QA is much higher than that of L-KYN metabolizing QA, making it easy for QA to accumulate in the brain region. Elevated QA levels can decrease dopamine, choline, and γ-aminobutyric acid (GABA) levels [108,109,110]. At the same time, continuous stimulation of NMDA receptors by QA can damage corresponding neuronal cells, leading to depression [111]. Nevertheless, ketamine, an NMDA receptor antagonist, can act on brain microglia and reduce the production of QA while antagonizing the effect of QA. In drug-resistant depression, baseline KYNA/QA can be used to predict ketamine response [112]. Nevertheless, ketamine is addictive, and it is limited to further use. In addition, hippocampal damage, which affects both neurons and glial cells, may be a mechanism of depression [113]. Hippocampal atrophy may be related to the imbalance of KYNA/QA [114]. Qualified people responsible for training (QPRT) have low activity in the hippocampus, and the hippocampus has difficulty metabolizing QA, increasing its susceptibility to damage. Wang et al. found that albiflorin can treat mouse models of olfactory bulbectomy (OBX), chronic unpredictable mild stress (CUMS), and lipopolysaccharide (LPS)-induced depression. Albiflorin normalizes metabolic dysregulation of phospholipid metabolism by inhibiting hippocampal cytoplasmic phospholipase A2 (cPLA2). In addition, inhibition of cPLA2 overexpression by albiflorin corrected the aberrant kynurenine pathway of tryptophan metabolism via the cPLA2-protein kinase B (Akt1)-indoleamine 2, 3-dioxygenase 1 regulatory loop and guided tryptophan catabolism to promote more significant hippocampal 5-HT biosynthesis [115]. Therefore, the above evidence suggests that the KYN pathway is crucial for depression and is a potential therapeutic target. It is thought that chronic inflammation causes over-activated IDO in the body and brain. Over-activated IDO leads to decreased KYN levels, producing excessive QA and less KYNA in the brain. The over-accumulation of QA damages neurons, leading to inflammation-induced depression. Maintaining KYNA-AQ balance is crucial for physiological homeostasis and preventing the development of depression.

### 4.3. Tryptophan Metabolism-Driven Therapeutic Drugs

COVID-19, glioma, and inflammation-induced depression are immune-mediated diseases connected to KYN pathway over-activation. Therefore, inhibiting IDO and TDO is a promising therapeutic modality. Inhibitors of IDO1 allow for restoring immune cell function. However, TDO affects immunosuppression via the TDO-L-KYN-AhR pathway. Thus, inhibiting TDO contributes to hindering immune escape [34]. In recent years, at least seven IDO1 inhibitors have been evaluated in Phase I or II clinical trials (Table 3), while TDO inhibitors are primarily in the preclinical stage. The chemical structures of five IDO1 inhibitors are publicly available, including indoles and heterocyclic aromatic hydrocarbons (indoximod and PF-06840003), hydroxylamines (Epacadostat), 4-phenylimidazoles (Navoximod), 1,2-diamino-substituted and 1-hydroxy-2-amino-substituted arylalkanes (KHK2455), and others (BMS-986205). IDO inhibitors such as Indoximod, Navoximod, Epacadostat, and BMS-986205 are well tolerated but not as effective as monotherapies and are therefore often studied in conjunction with immune checkpoint inhibitors [116].

In addition, research has been moving toward dual IDO/TDO inhibitors in recent years. Zhang et al. have developed dual IDO1/TDO inhibitors for treating inflammation-induced depression by inhibiting microglial cell activation, decreasing IDO1 expression, and decreasing pro-inflammatory cytokine and kynurenine levels in the mouse brain [117]. Zhang et al. found that sodium tanshinone IIA sulfonate (STS) can reduce the IDO1 and TDO2 enzymatic activities as an immunotherapeutic agent for colorectal cancer [118]. Liang et al. found that IDO1/TDO dual inhibitor RY103 shows greater preclinical efficacy in vivo than IDO1 selective inhibitor 1-L-MT [119]. In conclusion, it is necessary to explore the impact of IDO1 on cancer and search for novel IDO1 inhibitors and alternative therapeutic approaches to target IDO1.

**Table 3:** Currently investigated IDO1 inhibitors.

Molecules	Structure and Properties	Strategy	Major Findings	Active or Recruiting Studies
1-MT-L- Tryptophan 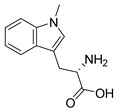	Non-specific competitive inhibitors Analogues of L-tryptophan Increase the level of KYNA in vivo	Single agent Pembrolizumab/nivolumab Temozolomide Adenovirus-p53 transduced dendritic cell (DC) vaccine	1-MT induces an increase in KYNA in vitro and in vivo. KYNA has immunomodulatory properties, and the transition to this branch of the canine uric acid pathway may be a potential mode of action for 1-MT [120].	Phase I/II: pediatric healthy (NCT03372239, NCT03852446), breast (NCT01042535, NCT01792050, NCT01302821), pancreatic (NCT02077881), prostate (NCT01560923), non-small cell lung cancer (NCT02460367), solid (NCT00739609, NCT00567931, NCT01191216), brain tumors (NCT05106296, NCT04049669, NCT02052648, NCT02502708), leukemia (NCT02835729), and melanoma (NCT03301636, NCT02073123)
1-MT-D- Tryptophan (indoximod) 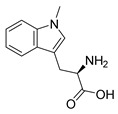	Prodrug: NLG802 Preferentially inhibit IDO2 Low in vitro activity but effective in vivo		NLG802 is widely absorbed and rapidly metabolized to indimod in all species tested, which is expected to increase the level of clinical drug exposure to indimod, thereby increasing therapeutic effects and expands the treatment population [117].	
Epacadostat INCB024360 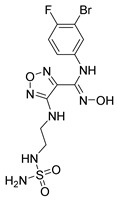	Selective reversible competitive inhibitor of IDO1 Antitumoral (decreases Tregs, increases synthesis of IFN-γ by T cells) but lack of activity as a monotherapy Metabolized by the intestinal microbiota and the enzyme UGT1A9 (AhR target)	Single agent Pembrolizumab Pembrolizumab/azacitidine Pembrolizumab/chemotherapy INCB001158/pembrolizumab Electroporation/pembrolizumab Nivolumab/Ipilimumab Nivolumab/chemotherapy Durvalumab Cladribine/cytarabine Idarubicin/cytarabine/ Dauno-Rubicin Rapamycin INCMGA00012 + RT + bevacizumab M7824 + BN-Brachyury + ALT-803 + Epacadostat (Immunotherapy) INCMGA00012, Epacadostat 600 mg BID, SV-BR-1-GM combination Intralesional SD101, Radiotherapy	The crystal structure of the complex of human indoleamine 2,3-dioxygenase 1 with its substrate tryptophan, inhibitor epacadostat, and/or effector indole ethanol is introduced. The structural characteristics of the active site that is crucial for substrate activation and the unique small molecule binding site have been revealed [121].	Phase I/II: peritoneal (NCT01982487, NCT02118285, NCT02042430, NCT02166905, NCT02785250, NCT02575807), thymic (NCT02364076), naso-pharyngeal (NCT04231864), gastric (NCT03196232), gastrointestinal (NCT03291054), pancreatic (NCT03432676, NCT03006302), non-small cell lung (NCT02862457, NCT02298153, NCT03402880, NCT03322566, NCT03322540), head and neck (NCT02178722, NCT03463161, NCT03823131), gastroesophageal (NCT03592407), metastatic colorectal (NCT03182894), and rectal cancer (NCT03516708), melanoma (NCT01961115, NCT01604889), genitourinary (NCT01685255), sarcoma (NCT03414229), solid tumor (NCT01195311, NCT02559492, NCT03361228, NCT03347123, NCT03085914, NCT02318277) Phase III: lung (NCT03348904) (NCT03347123), head and neck carcinoma (NCT03358472, NCT03342352), urothelial (NCT03361865, NCT03374488) and renal carcinoma (NCT03260894)
Linrodostat BMS-986205 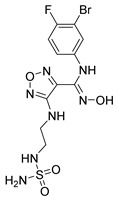	Selective, potent, and irreversible IDO1 inhibitor Restores T cell proliferation	Single agent Nivolumab Relatlimab/nivolumab Nivolumab/BCG Itraconazole/rifampin Nivolumab/chemotherapy Nivolumab/radiotherapy or Chemoradiotherapy Omeprazole	The combination of BMS-986205 and nivolumab is well tolerated in heavily pretreated patients, and the combination update safety and efficacy in advanced cancers across all tumor groups [122].	Phase I/II: liver (NCT03695250), advanced (NCT03792750, NCT03192943, NCT03459222), head and neck (NCT03854032) and bladder cancer (NCT03519256), malignancies multiple (NCT03346837), glioblastoma (NCT04047706), melanoma (NCT04007588, NCT02658890) Phase III: head and neck (NCT03386838), melanoma (NCT03329846), lung (NCT03417037) and bladder cancer (NCT03661320)
PF-06840003 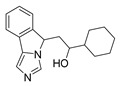	Non-competitive specific IDO1 inhibitors Oral use	Single agent	Studies have shown that PF-06840003 (up to 500 mg) has a long-lasting and well-tolerated pharmacological effect in patients with recurrent malignant gliomas [123].	Phase I/II: malignant gliomas (NCT02764151)
Navoximod, NLG-919, or GDC-0919 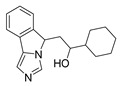	Reversible moderately selective noncompetitive inhibitor Synergy with indoximod Dose-dependent activation and proliferation of effector T cells Increases survival (± chemotherapy) Currently optimized by prodrug formulation	Single agent	Programmable co-delivery of the immune checkpoint inhibitor NLG919 and chemotherapeutic doxorubicin via a redox-responsive immunostimulating polymeric prodrug carrier. Systemic delivery of DOX via PSSN10 nanocarriers produces synergistic anti-tumor activity [124].	Phase I/II: solid tumors (NCT02471846, NCT02048709, NCT05469490)
KHK2455	Novel and selective oral IDO-1 inhibitor Long-lasting and potent activity	Avelumab Mogamulizumab	At all doses tested, KHK2455 in combination with mogamulizumab is well tolerated, safe, and dose-dependent; persistently suppresses KYN production; and shows early signals of anti-tumor activity [125].	Phase I/II: solid tumors (NCT02867007), urothelial carcinoma (NCT03915405), glioblastoma multiforme (NCT04321694)
LY-3381916 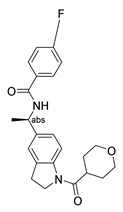	Potent and selective inhibitor of cell-based IDO1 activity Binding to newly synthesized apo-IDO1 lacking heme	Single agent or in combination with anti-programmed cell death ligand 1 (PD-L1) check-point antibody (LY3300054)	The central nervous system permeability of LY3381916 (rodent kp, uu 0.26) is significant. In addition, as an AhR agonist, a number of heme-binding IDO1 inhibitors have been shown to be a substitute for kynurenine. In addition, LY3381916 is shown to potentiate the activity of an anti-PD-L1 antibody (LY3300054) in a preclinical tumor model, which is associated with enhanced T cell responses [126].	Phase I/II: solid tumor, non-small cell lung cancer, renal cell carcinoma, triple negative breast cancer (NCT03343613)

Clinical trials can be accessed at https://www.clinicaltrials.gov/ (accessed on 2 October 2024).

## 5. Conclusions and Future Perspective

Tryptophan metabolism plays a crucial role in human health and disease. This review utilizes scientometric analysis to visually depict the comprehensive knowledge framework and current status of research about tryptophan metabolism, marking the first instance of such an analysis being conducted. Based on the analysis of the keyword co-occurrence network, keywords can be broadly categorized into four thematic clusters: cluster 1 (tryptophan metabolism pathway), cluster 2 (gut microbiota), cluster 3 (COVID-19 and glioma), and cluster 4 (inflammation-induced depression).

Following the above scientometric analysis results, this review carries out a detailed and comprehensive discussion. Tryptophan is mainly metabolized by the KYN, gut microbiota, and 5-HT pathways. The gut microbiota directly converts tryptophan to indole and its derivatives and regulates gut permeability, inflammation, and host immunity. Simultaneously, some gut microbial metabolites regulate tryptophan metabolism by activating specific receptors or enzymes. Moreover, tryptophan metabolites may serve as potential markers of clinical features such as inflammation, mental status, and prognosis and may guide clinical decision-making. The KYN pathway serves as the primary route of metabolism, and its enzymatic and receptor components present promising targets for treating inflammation and inflammation-induced diseases. The rate-limiting enzymes IDO and TDO can activate the KYN pathway, thereby counteracting the biological defense mechanism of severe infection, affecting the migration and invasion of glioma cells and promoting the development of COVID-19 and depression. Researchers are currently working on the development of IDO1 inhibitors to improve the efficacy of immunotherapy. Several small-molecule vaccine candidates (Indoximod, Navoximod, Epacadostat, and BMS-986205) are being evaluated in clinical trials. The imbalance between the metabolites KYNA and QA represents an imbalance in neuroprotection/neurotoxicity, affecting brain structure and function. In conclusion, research on tryptophan metabolism, particularly the KYN pathway, inflammation, tumor, and CNS diseases, has attracted widespread attention.

Although the mechanism of tryptophan metabolites binding to AhR receptors to regulate disease is precise, other potential mechanisms need to be explored. Drug development projects mainly focus on IDO1 and TDO, and research on enzymes, including KMO, KAT, and KYNU, need improvement. Evaluating the role and potential relationship of KYN metabolism-related enzymes and determining the correlation between metabolites is a promising direction for treating inflammation and nervous system disease. At the same time, the rationality of the systemic targeting of the KYN enzyme and its toxicity and efficacy for treating CNS disorders is under consideration. In addition, most current studies are based on one pathway of tryptophan metabolism, ignoring the dynamic equilibrium relationship and interaction of the three pathways. Studying the above issues will help to understand the disease mechanism better, identify key regulatory sites, and quickly develop drugs to prevent and treat diseases.

## Figures and Tables

**Table 1:** Details of the keyword co-occurrence network.

Hotspot	Cluster	Keyword	Centrality	Frequency
**Tryptophan metabolism pathway**	1	tryptophan	518.51	1
	1	serotonin	84.38	4
	1	quinolinic acid	43.97	12
	1	kynurenine pathway	41.05	8
	1	indoleamine 2,3-dioxygenase	14.66	19
	1	melatonin	5.63	17
	1	tryptophan 2,3-dioxygenase	3.44	35
	1	autism	3.39	39
	1	IDO1	0.00	42
	1	kynurenines	0.00	43
	1	niacin	0.00	15
	1	nicotinamide	0.00	21
	1	rat	0.00	34
	1	NAD	0.00	53
**Gut microbiota**	2	tryptophan metabolism	342.86	2
	2	kynurenine	164.30	3
	2	metabolism	26.09	11
	2	gut microbiota	20.34	5
	2	metabolomics	18.65	9
	2	aging	4.57	18
	2	chronic kidney disease	3.48	41
	2	anti-inflammatory	2.36	47
	2	central nervous system	1.20	48
	2	microbiota	0.93	32
	2	ulcerative colitis	0.72	23
	2	positron emission tomography	0.54	33
	2	hippocampus	0.18	29
	2	plasma	0.18	54
	2	gut microbiome	0.00	28
	2	mass spectrometry	0.00	31
**COVID-19 and glioma**	3	COVID-19	9.52	49
	3	glioma	7.83	24
	3	aryl hydrocarbon receptor	6.97	16
	3	microbiome	4.14	44
	3	colitis	3.69	27
	3	indole	2.69	30
	3	3-dioxygenase	2.09	36
	3	indoleamine 2	1.41	25
	3	tryptophan 2	0.35	55
**Inflammation-induced depression**	4	inflammation	88.80	7
	4	depression	29.04	6
	4	kynurenic acid	18.94	10
	4	irritable bowel syndrome	10.62	52
	4	neopterin	8.94	20
	4	oxidative stress	7.58	26
	4	anxiety	5.43	22
	4	interferon-gamma	3.06	51
	4	3-hydroxyanthranilic acid	2.25	46
	4	IDO	1.62	14
	4	L-tryptophan	1.11	13
	4	cytokine	1.07	50
	4	3-hydroxykynurenine	0.24	37
	4	stress	0.18	45
	4	amino acids	0.00	38

**Table 2:** The highly cited papers related to tryptophan metabolism.

Rank	Times Cited	The Title of Article	Year	Journal			
				Name	Country	Impact Factor (2022)	Main Conclusions
1	760	CARD9 impacts colitis by altering gut microbiota metabolism of tryptophan into aryl hydrocarbon receptor ligands [19]	2016	Nature Medicine	USA	82.9	CARD9 alters intestinal inflammation, metabolites, and their microbial composition by affecting the binding of intestinal tryptophan metabolites to AhR [19].
2	243	Increased Tryptophan Metabolism Is Associated with Activity of Inflammatory Bowel Diseases [20]	2017	Gastroenterology	USA	29.4	Tryptophan has a high degradation activity in active IBD patients, with a significant increase in metabolic product levels, such as QA. In addition, tryptophan deficiency may lead to the development of IBD [20].
3	186	Microbiota-related Changes in Bile Acid & Tryptophan Metabolism are Associated with Gastrointestinal Dysfunction in a Mouse Model of Autism [21]	2017	EBioMedicine	Netherlands	11.1	Bacterial metabolism in intestinal bile metabolism is deficient in BTBR mice, and Bifidobacterium bifidum and Blautia species are reduced. Changes in gut microbiota affect gastrointestinal function and social activities in BTBR mice [21].
4	67	Tryptophan metabolism drives dynamic immunosuppressive myeloid states in IDH-mutant gliomas [22]	2021	Nature Cancer	Germany	22.7	A target for immunotherapy of IDH-mutant tumors is identified: tryptophan metabolism. Evidence is provided for the existence of an intratumoral network of resident and recruited myeloid cells that are dependent on the glioma genotype [22].
5	66	Microbiota tryptophan metabolism induces aryl hydrocarbon receptor activation and improves alcohol-induced liver injury [23]	2021	Gut	England	24.5	A new therapeutic target involving gut microbiota in the AhR pathway has been identified for treating alcoholic liver disease [23].
6	54	Fuzhuan Brick Tea Polysaccharide Improved Ulcerative Colitis in Association with Gut Microbiota-Derived Tryptophan Metabolism [24]	2021	Journal of Agricultural and Food Chemistry	USA	6.1	FBTP may alleviate UC by regulating microbial metabolism and gut microbiota and repairing gut barrier [24].
7	23	Honeybee gut Lactobacillus modulates host learning and memory behaviors via regulating tryptophan metabolism [25]	2022	Nature Communications	Berlin	16.6	Host-specific Lactobacillus strains promote memory behavior by converting tryptophan into indole derivatives that activate the AhR of the host [25].
